# Effects of *Smallanthus sonchifolius* Flour on Metabolic Parameters: A Systematic Review

**DOI:** 10.3390/ph17050658

**Published:** 2024-05-20

**Authors:** Isabela Frazão da Silva, Wesley Rossi Bragante, Renato Cesar Moretti Junior, Lucas Fornari Laurindo, Elen Landgraf Guiguer, Adriano Cressoni Araújo, Adriana M. R. Fiorini, Claudia C. T. Nicolau, Marie Oshiiwa, Enzo Pereira de Lima, Sandra Maria Barbalho, Luís R. Silva

**Affiliations:** 1 Department of Biochemistry and Nutrition, School of Food and Technology of Marília (FATEC), Marília 17500-000, São Paulo, Brazil; 2 Department of Biochemistry and Pharmacology, School of Medicine, Universidade de Marília (UNIMAR), Marília 17525-902, São Paulo, Brazil; 3 Department of Biochemistry and Pharmacology, School of Medicine, Faculdade de Medicina de Marília (FAMEMA), Marília 17519-030, São Paulo, Brazil; 4 Postgraduate Program in Structural and Functional Interactions in Rehabilitation, School of Medicine, Universidade de Marília (UNIMAR), Marília 17525-902, São Paulo, Brazil; 5 UNIMAR Charitable Hospital, Universidade de Marília (UNIMAR), Marília 17525-902, São Paulo, Brazil; 6 CICS-UBI—Health Sciences Research Centre, University of Beira Interior, 6201-001 Covilhã, Portugal; 7 SPRINT—Sport Physical Activity and Health Research & Innovation Center, Instituto Politécnico da Guarda, 6300-559 Guarda, Portugal; 8 CERES, Department of Chemical Engineering, University of Coimbra, 3030-790 Coimbra, Portugal

**Keywords:** yacon flour, *Smallanthus sonchifolius*, glycemia, lipids, metabolic syndrome, oxidative stress, inflammation

## Abstract

*Smallanthus sonchifolius*, popularly known as yacon, is a member of the *Asteraceae* family. Due to its medicinal and edible value, yacon is consumed by different populations. Yacon is unique due to its high fructo-oligosaccharide and inulin content, as well as flavonoids, sesquiterpene lactones, and phenolic acids. Roots can be used to produce flour, which is less perishable and can be applied in various industrial products. This systematic review focuses on the effects of yacon flour on metabolic parameters. PubMed, Cochrane, Embase, Science Direct, Scopus, Web of Science, and Google Scholar databases were consulted, and PRISMA guidelines were followed in the selection of the studies. In total, 526 articles were found in the databases, and of these, only 28 full texts were eligible for inclusion. After applying the inclusion and exclusion criteria, seven studies were finally included. The results showed that the use of yacon flour can reduce glycemia, HbA1c, advanced glycation ends, plasma lipids, body fat mass, body weight, and waist circumference and improve intestinal microbiota and the antioxidant status. Further exploration of the effects of yacon flour is warranted, and additional clinical trials are necessary to determine the optimal daily consumption levels required to assist in improving metabolic parameters.

## 1. Introduction

*Smallanthus sonchifolius,* popularly known as yacon, is an herbaceous plant belonging to the Asteraceae family that is native to the Andean region range extending from Colombia to northwestern Argentina. It grows in subtropical and warm temperate environments at altitudes ranging from 600 to 3500 m [1]. *S. sonchifolius* is a perennial plant whose aerial stems are cylindrical and greenish in color, have hairiness over the entire surface, and can measure up to 2.5 m in height, exhibiting tuberous roots, similar to fruits, of different shapes and sizes, possessing a sweet and pleasant flavor. The plant reaches physiological maturity after 6 to 10 months of planting, and the roots are harvested after 10 to 12 months [2,3]. Due to its medicinal, commercial, and edible value, yacon has been consumed by different populations in fresh form, in salads, food preparations, juices, syrups, and fermented beverages, and as flour for a long time [4,5,6,7,8].

Yacon root has unique importance due to its high content of fructo-oligosaccharides (FOS) (between 40% and 70% of the carbohydrates) and inulin, in addition to containing significant amounts of flavonoids, sesquiterpene lactones, and phenolic acids [9,10]. Therefore, it is used as a source of prebiotics in different food products and can have beneficial effects for health [11,12]. Inulin and FOS also have probiotic properties, which stimulate the activity and growth of intestinal bacteria, such as *Bifidobacteria* [8,13,14]. Additionally, the inulin present in yacon can help regulate glycemia. Animal studies also suggest that yacon may have a protective effect on pancreatic beta cells. It can also act as a natural appetite suppressant, helping control hunger and reduce caloric intake [15,16,17].

The starchy roots contain minerals, such as zinc, phosphorus, iron, copper, calcium, some vitamins from the B complex and vitamin C, tryptophan, proteins, lipids, and polyphenols (such as protocatechuic, chlorogenic, caffeic, and ferulic acids), in addition to the aforementioned high amounts of indigestible oligosaccharides and inulin. Other bioactive compounds may include sterols, diterpenes, fatty acids, volatile oils, *p*-hydroxyacetophenone, and octulosonic acid derivatives that bring benefits in Alzheimer’s disease and can reduce blood lipid levels, protecting the kidneys and the liver [9,18,19,20]. Yacon rhizomes, leaves, and extracts exhibit large amounts of phenolic acids, mainly caffeic acid and its derivatives. The presence of these bioactive compounds confers strong antioxidant and anti-inflammatory power. In addition, these substances can diminish glycemia and improve insulin sensitivity. The leaves possess sesquiterpene lactones on the surface of glandular trichomes. These compounds demonstrate the ability to impede the proliferation of cancer cells across multiple cancer types, such as cervical, glioma, and colorectal cancers. Additionally, they exhibit antimicrobial properties by effectively inhibiting Gram-positive bacteria [12,21,22,23,24,25,26,27,28].

The use of yacon leaves can reduce glycemia in diabetic rats (streptozotocin-induced diabetes) and can elevate insulin levels and sensitivity. Furthermore, the leaf extract can inhibit the action of the enzyme diphenyl peptidase 4 [25,29,30].

Phenolic acids and melampolide are the primary compounds found in yacon leaves, and sesquiterpene lactones mainly accumulate in this part of the plant. Enhydrin and uvedalin are the most representative sesquiterpene lactone types. Enhydrin compounds are active compounds and can be considered antidiabetic agents [29,31]. Of the 10 sesquiterpene lactone components found in yacon leaves, enhydrin and uvedalin are unique in inhibiting nuclear factor kappa-B (NF-kB), which is related to the regulation of the immune system and inflammatory processes [32,33].

**Figure 1:**
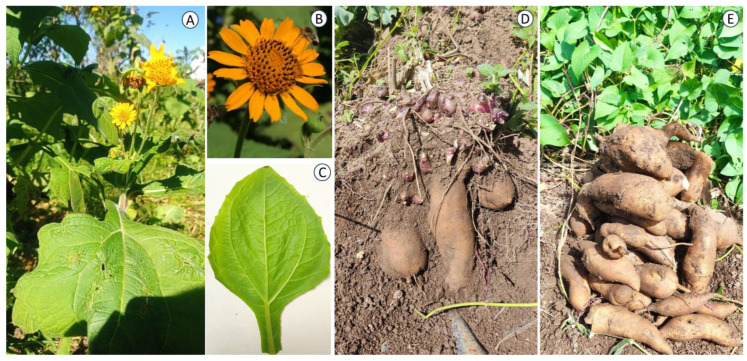
Yacon plant. (**A**) Whole plant, (**B**) flower, (**C**) leaf, and (**D**,**E**) yacon rhizomes.

**Figure 2:**
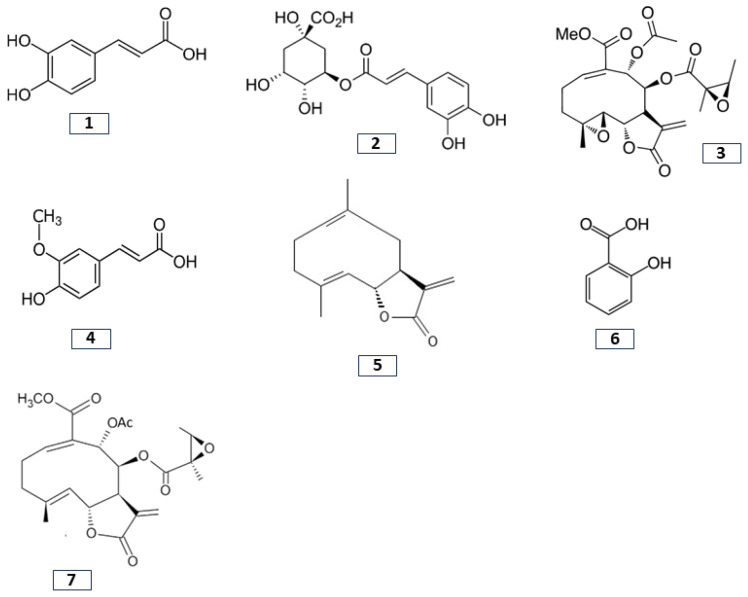
Main bioactive compounds found in yacon. **1**: Caffeic acid; **2**: chlorogenic acid; **3**: enhydrin; **4**: ferulic acid; **5**: melampolide; **6**: phenolic acid; and **7**: uvedafolin.

The presence of the different bioactive compounds isolated from yacon is associated with the neutralization of free radicals that are related to oxidative stress, inflammation, and cellular damage and could induce chronic diseases, such as cardiovascular disease (CVD) and cancer [26,34]. Figure 1 shows the different parts of the yacon plant, and Figure 2 shows the main bioactive compounds. Table 1 shows the main effects of the bioactive compounds present in this plant.

Additionally, some studies suggest that yacon may help protect the liver from damage caused by toxic substances, also due to the antioxidant and anti-inflammatory properties [8,49]. In addition to its health benefits, yacon has a low energy value and a sweet taste. It can be used as a potential alternative sweetener to sucrose and dietary products in the food industry. Moreover, it can be used to prepare different and innovative food products [29,50,51].

Since the modern lifestyle has led to an exponential increase in obesity, diabetes, dyslipidemia, and metabolic syndrome, as well as CVD and cancer, being among the leading causes of death worldwide, a change in eating habits and the consumption of healthier products can help prevent and even treat these diseases [52,53]. Due to the numerous health benefits of yacon and the lack of a review showing the effects of yacon flour, this work aims to systematically review the effects of yacon root flour on metabolic parameters related to metabolic syndrome. To the best of our knowledge, this is the first review of the effects of yacon flour on metabolic parameters.

## 2. Materials and Methods

### 2.1. Focal Question

This systematic review was carried out to answer the following question: “Can yacon root flour benefit metabolic parameters?”

### 2.2. Language

Only studies published in English were selected.

### 2.3. Databases

This review included studies published in the PubMed, Cochrane, Embase, Science Direct, Scopus, Web of Science, and Google Scholar databases.

The descriptors used were as follows: yacon or *Smallanthus sonchifolius* or yacon flour or yacon root flour and glycemia or insulin or insulin resistance or diabetes or antioxidant or anti-inflammatory or obesity or blood pressure or lipids or dyslipidemia or obesity or body weight or metabolic syndrome or cardiovascular disease or intestinal microbiota.

**Figure 3:**
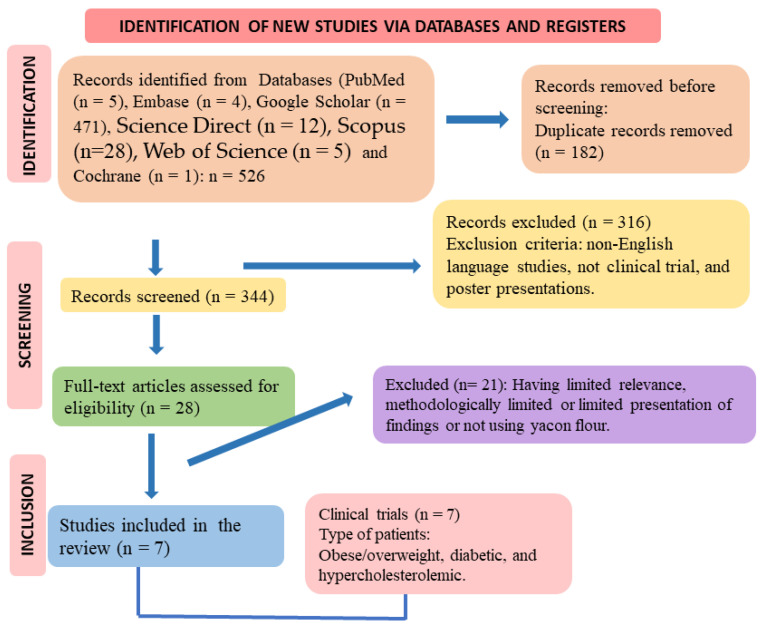
The flowchart shows the selection of studies (according to PRISMA guidelines).

The use of these descriptors helped identify studies related to yacon potatoes and their health effects. The Preferred Reporting Items for a Systematic Review and Meta-Analysis (PRISMA) guidelines [54,55] guided the search for studies. Figure 3 shows the flowchart of study selection.

### 2.4. Study Selection

Human interventional studies were used as the inclusion criterion for clinical trials. We also included studies performed on the intestinal microbiota, inflammation, and oxidative stress since they are closely linked to obesity, diabetes, and other metabolic changes. The exclusion criteria were studies not published in English, reviews, editorials, letters to editors, conferences, case reports, and poster presentations.

### 2.5. Data Extraction

The search period included studies published in the past 10 years. Population, Intervention, Comparison, and Outcomes (PICO) format was followed to evaluate and extract the data.

### 2.6. Search and Inclusion of the Relevant Articles

Study selection was according to the Preferred Reporting Items for a Systematic Review and Meta-Analysis (PRISMA) guidelines [54,55].

### 2.7. Quality Assessment

*The Cochrane Handbook* was followed to perform risk bias evaluation [56].

## 3. Yacon and Health Effects

### 3.1. Yacon Root Flour

**Figure 4:**
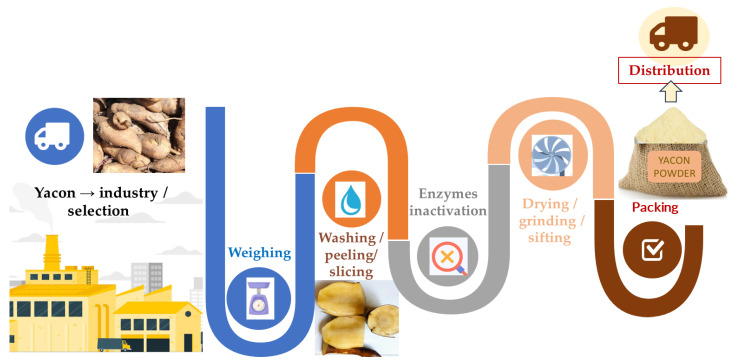
The main steps in yacon flour production and commercialization.

**Figure 5:**
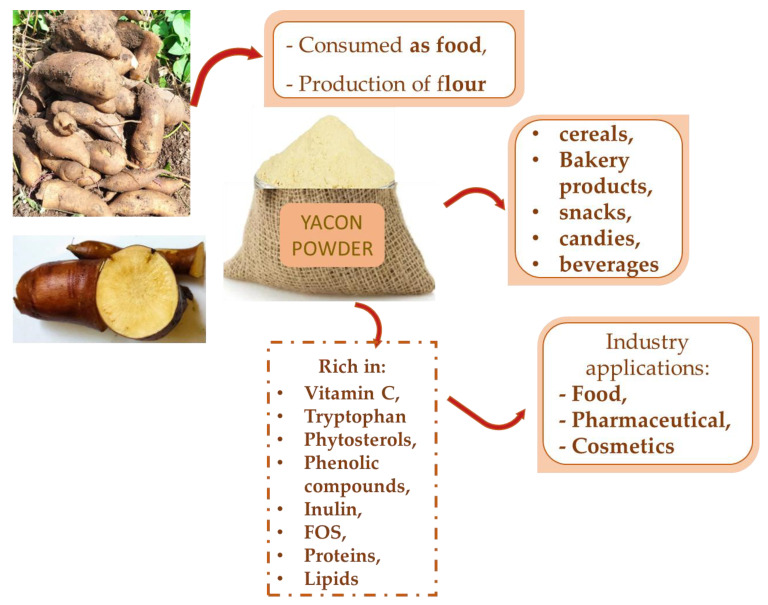
Yacon roots can be transformed into flour that possesses many bioactive compounds and can be applied to several products. FOS: fructo-oligosaccharides.

Yacon roots can be used to produce flour, a less perishable product that can be applied to numerous industrial products. The flour can be stored and conserved for longer time, in addition to being easy to add to several products [57]. In addition, yacon flour is a sweet-flavored ingredient with a low caloric value, making it an option for diabetics or other consumers with the aim to follow a healthier diet. Its relevant nutritional properties make this plant an important raw material for the dietary product market for the general population and those with diabetes and other metabolic risk factors. The substitution of sugar with yacon results in a low-calorie product with satisfactory sensory features [1,14,58,59,60]. The production of yacon flour (Figure 4) propitiates the development of bakery products, encouraging the use and ingestion of functional, healthier products. For example, the flour can be used in the production of breads, cakes, cupcakes, biscuits, candies (as a gelling agent), and snacks and as a sugar substitute (Figure 5). These products can be claimed to be low in calories, rich in fibers, and a source of minerals, vitamins, and other nutrients [59,61,62]. The consumption of products with these characteristics can assist in reducing glycemia and blood lipid levels and prevent constipation, obesity, and other metabolic risk factors [61,63,64].

In a study using yacon root flour for bread production, three loaves were prepared with a yacon root flour concentration of 5%, where the first formulation was composed of pulp flour, the second of rind flour, and the third of rind flour and potato pulp. Results were obtained by ordering, preference, and sensory analysis tests for texture and flavor attributes, demonstrating no significant difference between the formulations. The results obtained in this study show the potential for commercializing this product. Furthermore, flour is an ingredient that is easy to apply to many different products and adds a high nutritional value [11].

### 3.2. Antioxidant and Anti-Inflammatory Effects of Yacon Flour: In Vivo Evidence

Due to its many bioactive compounds, yacon can have anti-inflammatory and antioxidant properties. In vivo studies have revealed these activities.

A study on Wistar rats investigated the effects of yacon flour on inflammation and oxidative stress in animals with induced colorectal cancer (CRC). Wistar male rats were divided into four groups and maintained for 8 weeks: S (basal diet, *n* = 10), Y (yacon flour + basal diet, *n* = 10), C (CRC-induced control + basal diet, *n* = 12), and CY (CRC-induced animals + yacon flour, *n* = 12). CRC was induced by intraperitoneal injections of 1,2-dimethylhydrazine (25 mg/kg body weight). Y and CY groups were supplemented with 7.5% of the prebiotic FOS from yacon flour. The results showed that yacon flour lowered lipopolysaccharides, TNF-α, and IL-12 but had no effects on oxidative stress. Additionally, yacon flour benefited alterations provoked by CRC induced with 1,2-dimethylhydrazine in Wistar rats. Cancer increased TNF-α levels and SCFA production, while decreasing total antioxidant capacity. However, using yacon flour decreased intraluminal pH, TNF-α, and the TNF-α/IL-10 ratio and augmented secretory immunoglobulin A, suggesting its potential in maintaining intestinal health and mitigating CRC damage in animal models [8].

An investigation using a combination of yogurt (with two strains of probiotics) and yacon flour was performed in Swiss mice fed a high-fat or standard diet. The results revealed that this combination produced positive effects since it significantly reduced glycemia, body weight, and triacylglycerol levels. Moreover, it significantly decreased peri-epididymal fat accumulation (−44.2%; *p*  <  0.05) and inflammatory biomarkers and improved insulin signaling [63].

In another study, the researchers showed that Wistar rats treated with a high-fat diet and with yacon or yacon flour (340or 680 mg FOS/kg/d, respectively) had markedly reduced serum pro-inflammatory cytokine levels, and animals in the second group had reduced macrophage infiltration and MCP-1 expression in the visceral adipose tissue, associated with higher pAkt/Akt expression [65].

Higashimura and colleagues investigated the effects of a yacon-containing diet on the intestinal environment in mice, including microbial composition, short-chain fatty acid levels, and mucus content. The results indicated that the mice had considerably higher lactic acid, succinic acid, acetic acid, and propionic acid concentrations. The fecal mucin content was also higher in mice receiving the yacon-containing diet. The results showed the effects of yacon administration on intestinal inflammation using the 2,4,6-trinitrobenzene sulfonic acid-induced colitis mouse model and suggested that oral ingestion of yacon root alters the intestinal environment, therefore inhibiting intestinal inflammation [66].

The effects of yacon flour and inulin in high-fed rats showed that the flour primarily promotes effects by reducing visceral white adipose tissue expansion and downregulating hypoxia-inducible transcription factor-1α (HIF-1-α). Furthermore, it improved the antioxidant system; decreased lipid peroxidation and the expression of pro-inflammatory TNF-α, IL-1β and IL-6, MCP-1, and transforming growth factor-β1 (TGF-β1); and elevated anti-inflammatory IL-10. The administration of inulin could improve visceral white adipose tissue pathology and inhibited inflammatory processes and oxidative stress, mainly by augmenting glutathione levels and decreasing IL6 expression [67].

Alemmán et al. [68] investigated the effect of yacon flour on Wistar rats fed a standard diet with the incorporation of a 10% fructose solution. After 20 weeks, the animals were fed yacon flour (340 mg FOS/kg) or fenofibrate (30 mg/kg) for 16 weeks. The results showed a significant decrease in lipid levels in plasma, body weight gain, transaminase activities, and improved insulin response. In the liver, the flour reduced the fructose-induced steatosis and inflammatory process. Moreover, total collagen deposition was reduced, as was TGF-*β*1 mRNA expression (about 78%) and also the nuclear localization of *p*-Smad2/3. Yacon flour also significantly reduced the activation of resident hepatic stellate cells, suggesting that yacon could be considered to reduce liver damage resulting from a high-fructose diet consumption and could be a nutritional strategy in metabolic-associated fatty liver disease management.

**Figure 6:**
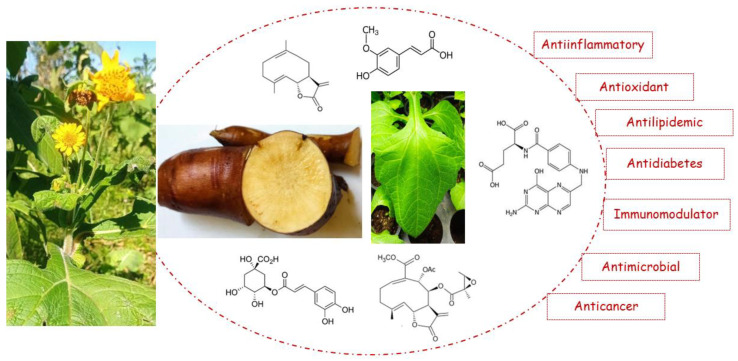
Main bioactive compounds present in yacon and its health benefits.

Other authors have investigated the effects of yacon flour (340 mg fructo-oligosaccharide/kg/day/90 days) on lipid peroxidation and other parameters of oxidative stress in both kidneys and liver of diabetic Wistar rats and observed a significant reduction in the levels of malondialdehyde in both organs. Hepatic and kidney catalase and superoxide dismutase activities were significantly reduced in diabetic Wistar rats compared with controls. There was also an increase in the levels of glutathione and glutathione peroxidase in the liver and kidneys. These results may indicate that yacon flour can be used to reduce oxidative stress and prevent the consequences of this condition [69]. Some of the health benefits of yacon are shown in Figure 6.

### 3.3. Hypoglycemic and Hypolipidemic Effects of Yacon Flour: In Vivo Evidence

Although several studies have shown the benefits of yacon in reducing glycemia and plasma lipids, not many authors have investigated the effects of yacon flour on this metabolic imbalance. Next, we describe the studies that have investigated yacon flour as a hypoglycemic or a lipid-lowering agent.

Honoré et al. [65] showed that animals fed with a high-fat diet supplemented with yacon or yacon flour (340 or 680 mg FOS/kg/d, respectively) had significantly reduced weight gain and showed a reduction in the visceral fat pads. In addition, the animals showed a dose-dependent improvement in the lipid profile and atherogenic index. Furthermore, the rats fed yacon flour with 680 mg of FOS showed reduced glycemia and improved insulin levels, glucose tolerance, and insulin sensitivity. The authors also observed downregulation in the expression of many adipocyte-specific transcription factors, such as CCAAT/enhancer-binding protein a (C/EBP-a), peroxisome-proliferator-activated receptor gamma2 (PPAR-γ2), and activating protein (aP2) mRNA levels. In conclusion, the consumption of yacon flour showed anti-obesity effects due to the inhibition of adipogenesis and the improvement of visceral adipose tissue function.

The use of yacon flour was also investigated at different concentrations (5–15%) in Wistar rats, and the results demonstrated that postprandial glycemia was reduced at different glucose peaks in these rats compared to the control group, especially at 15% concentration. Furthermore, yacon flour consumption increased fecal fat excretion in proportion to the amount of flour added to the diet [70].

Another study evaluated the phytochemical profile of methanolic extracts from the leaves of 14 yacon varieties, and the results showed that they were rich in antioxidants (polyphenols, tannins, and flavonoids). Results also showed anticholinesterase and antidiabetic activity (by inhibiting α-amylase and α-glucosidase), which could act as adjuvants in the therapeutic approach to Alzheimer’s disease and diabetes. Antioxidant activity has also been observed through tests such as FRAP, 2,2-diphenyl-1-picryl hydrazyl (DPPH), nitric oxide (˙NO), and superoxide (O_2_˙^−^) scavenging and lipid peroxidation inhibition assays [71].

According to another study, the use of yacon flour (340 or 6800 mg FOS/kg/day) in Wistar rats with diabetes induced by streptozotocin did not significantly alter rat body weight, but there was a significant reduction in serum triglycerides and VLDL-c. In addition, the authors observed an increase in the insulin-positive cell mass distributed in small cell clusters in the pancreas exocrine parenchyma. With regard to insulin, they observed only a slight increase in plasma. GLP-1 levels in the cecum were significantly higher in flour-fed animals than in controls. The study concluded that these effects suggest that this hormone may be the main mediator of the lipid-lowering actions of FOS. Given these findings, it is possible that yacon flour has multiple benefits in diabetes-associated dyslipidemia [72].

As mentioned before, the combination of yogurt and yacon flour administered to Swiss mice fed a high-fat or standard diet significantly reduced glycemia, body weight, and triglyceride levels. The authors also observed a significant reduction in peri-epididymal fat accumulation and an improvement in insulin signaling [63].

In another in vivo study, Wistar rats were divided into three groups (non-diabetic controls, diabetic controls, and diabetic rats treated with yacon). The treated group received a yacon flour tablet (340 mg fructo-oligosaccharide/kg/day/90 days), showed no significant variation in plasma glucose levels, but, interestingly, showed a significant increase in fasting plasma insulin levels. However, this increase was not sufficient to maintain blood glucose levels within normal limits. The results of the study indicate that yacon root flour is a potential dietary supplement with high antioxidant activity in vivo, with a response under plasma insulin. Plasma cholesterol and triacylglycerol levels and liver fatty acid composition, which were altered in diabetic rats, were restored to near-normal levels by yacon treatment [69].

In Wistar rats fed a high-fat diet and treated with goat yogurt supplemented with yacon flour, some authors [73] showed that the consumption led to a reduction in the body mass index, body weight, fasting glycemia, homeostasis model assessment of insulin resistance (HOMA-IR), and the atherogenic index.

The aim of another study was to evaluate the effects of subchronic oral consumption (twice daily for four months) of yacon root flour in Wistar rats. Consumption of the flour did not reduce glycemia in normal rats but resulted in a significant decrease in triglyceride levels [74].

Oliveira et al. [75] investigated the effects of using a solution prepared with 30% yacon (1.2 g of freeze-dried yacon) in diabetic (streptoziton-induced) rats (acute and chronic administration). The results showed that the freeze-dried product resulted in the lowest average weight values when compared to the average glycemia values of the different groups; the yacon diabetic group also showed a hypoglycemic response. With chronic administration, there was no significant difference in the mean weight in the different groups, but there was a significant difference in glycemia between the groups. HbA1c levels showed no difference, and food intake was lower in the yacon diabetic group.

### 3.4. Metabolic Effects of Yacon Flour in Humans: The Results of Clinical Trials

Table 2 shows the clinical trials related to the use of yacon flour in human metabolic disorders.

One interesting study showed the effects of yacon flour consumption and an energy-restricted diet on the intestinal microbiota of overweight adults, and the authors evaluated fecal samples on the first and last days of the study. They also evaluated 16S rRNA sequencing to investigate the effects of yacon fermentation on the intestinal microbiota composition. Although the results of the study are interesting, the sample size was small and losses at the end of the study were significant. These facts may be potential biases in the interpretation of the study results [61].

Another study aimed to investigate the effect of yacon powder on HbA1c levels in elderly people. The results showed that the use of yacon powder is statistically significant in reducing serum glucose levels in the elderly, clearly indicating its potential benefit in combating one of the main factors related to metabolic syndrome. However, several biases must be taken into account, such as the small sample size, the short duration of the study, and the lack of blinding in the interpretation of results [76]. 

In another trial, the authors studied the effects of yacon flour on biochemical markers, such as advanced glycation ends (AGEs), anthropometric variables, and body composition. A positive effect was observed, such as a decrease in AGEs, which helps reduce pro-inflammatory cytokines and thus prevent chronic metabolic problems, as well as a reduction in body measurements, favoring a reduction in the severity of metabolic problems. However, the sample size was small, and the age range of the participants was wide. The amount of yacon flour used in the study can be considered small, and the participants were also subjected to a calorie-restricted diet, which are possible biases in the study [64].

Another study evaluated the influence of yacon flour on antioxidant capacity and oxidative stress. Although the results are important in showing that the flour can reduce oxidative stress, the participants were subjected to a calorie-restricted diet, which may have interfered with the results; the number of participants was small, which is a possible bias in this study [60].

The effects of yacon flour were also investigated in 26 overweight adults who were instructed to follow an energy-restricted diet and consumed a shake containing 25 g of yacon flour (0.1 g of FOS/kg body weight). The authors evaluated biochemical parameters, such as glycemia, insulin, lipid, and the liver function profile; anthropometric parameters; body composition; blood pressure; and intestinal function. The results showed that the consumption of the shake does not provoke adverse gastrointestinal events and reduces the waist circumference, body weight, sagittal abdominal diameter, waist-to-height index, and body fat. Although these results are promising, the small size of the sample could be a bias in interpreting the results [77].

Yacon flour was also used to formulate a shake, in the belief that it had the potential to prevent chronic diseases, mainly due to its positive effect on the glycemic response. However, no evident results were obtained in terms of the glycemic response proposed by the study. Additionally, the short duration of the trial, the small sample size, and the lack of report on the side effects introduce some biases into the interpretation of the results. Furthermore, the fact that it was not a double-blind trial may have compromised the fidelity of the result interpretation [78].

One important study demonstrated the significance of yacon powder consumption in reducing biomarker levels of glucose, lipids, and triglycerides, showing that yacon can be considered in the prevention of diabetes and dyslipidemia. However, the short duration of the study, the small number of volunteers, and the lack of specification regarding sex may compromise the interpretation of the results [79].

Table 3 presents the potential bias assessment of the clinical trials included in the study.

## 4. Conclusions

As shown in the aforementioned studies, yacon flour is promising as a coadjuvant in preventing alterations in the body weight, waist circumference, body fat mass, glycemia, HbA1c, and dyslipidemia. It may also reduce AGEs and improve antioxidant capacity, thereby reducing oxidative stress and inflammatory processes. No alterations were observed in diastolic and systolic blood pressure after the use of yacon flour. However, the effects of using yacon flour need to be further explored, and more clinical trials should be carried out to identify the amounts of flour that should be consumed daily. Furthermore, products prepared with yacon flour should be explored and investigated in clinical trials to consider whether they may also help with weight loss, blood glucose and lipid control, oxidative stress reduction, and inflammation reduction.

## Figures and Tables

**Table 1:** Main bioactive compounds found in *Smallanthus sonchifolius* roots and leaves.

Bioactive Compound	Biological Effects	References
Caffeic acid	Metabolic effects: antidiabetic; other effects: antidepressant, anticancer, and antiviral	[35,36]
Chlorogenic acid	Metabolic effects: anti-inflammatory, antioxidant, and antidiabetic	[37,38]
Enhydrin	Metabolic effects: antidiabetic and antioxidant; other effects: antimicrobial	[39,40]
Ferulic acid	Metabolic effects: antidiabetic; other effects: antimicrobial and anti-carcinogenic	[41,42]
Melampolide	Metabolic effects: antioxidant and reduces angiogenesis; other effects: antimicrobial, inhibition of DNA synthesis, and increased cell apoptosis	[43,44]
Phenolic acid	Metabolic effects: anti-inflammatory, antidiabetic, and antioxidant; other effects: antimicrobial and anticancer	[45,46]
Uvedafolin	Metabolic effects: antidiabetic and anti-inflammatory; other effects: anti-bacterial (against *Anthracis* and MRSA) and anticancer	[47,48]

**Table 2:** Studies showing the effects of yacon root flour on metabolic parameters.

Reference	Model/Country	Population	Intervention/Comparison	Outcomes	Adverse Effects
[61]	Randomized, parallel, double-blind, placebo-controlled, 6-week clinical trial/Brazil	21 overweight adults	Participants consumed a drink containing 25 g of yacon flour (*n* = 11) or a placebo (*n* = 10) and a prescription for an energy-restricted diet at breakfast.	Yacon group: elevation in *Bifidobacterium*, *Blautia*, *Subdoligranulum*, and *Streptococcus* and a positive correlation between SCFAs versus *Coprococcus* and *Howardella* and a negative correlation between AGEs and early glycation products versus the genera *Ruminococcus* and *Prevotella*.	NR
[76]	Randomized, 8-week clinical trial/Pakistan	20 diabetic, obese elderly, ? ♂, ? ♂, age ? ± ? y, BMI ? ± ? kg/m^2^	Participants in the yacon group (G1) received individual sachets of 20 g of yacon powder with 8 g of FOS twice a day.	Long-term yacon powder use: considerable decrease in body weight, body fat, waist circumference, and blood glucose levels.	Minimal digestive pain
[64]	Randomized, parallel, double-bind, placebo-controlled clinical trial/Brazil	26 mildly hypercholesterolemic participants, 11 ♂, 15 ♀, BMI 30.44 ± 2.46 kg/m^2^, age 35 ± 8.54 y	Participants were allocated to the control group (*n* = 13) or the yacon flour group (*n* = 13; breakfast drink containing either 25 g of yacon flour or 8.7 g of FOS).	AGEs and early glycation products did not increase in the yacon flour group. Soluble receptors for AGEs (sRAGEs) decreased regardless of the group. Modifications in AGEs were positively related to changes in body fat.	NR
[60]	Randomized, double-blind, parallel, placebo-controlled, 2-arm clinical trial/Brazil	26 overweight adults, ? ♂, ? ♂, age 20–45 y, BMI 30.4 ± 2.4 kg/m^2^	Participants were allocated to the control group (*n* = 13) or the yacon flour group (*n* = 13; received a drink with 25 g of yacon flour/6 w). Intestinal permeability, fecal SCFAs, oxidative stress, and inflammatory markers were evaluated in vivo.	The yacon flour group showed an elevation in the plasma antioxidant capacity, a reduction in oxidative stress (measured by the activity of catalase and glutathione S-transferase; malondialdehyde, nitric oxide, and antioxidant capacity), and SCFAs in adults with obesity or overweight.	Flatulence and abdominal pain (yacon flour group)
[77]	Randomized, double-blind, parallel, placebo-controlled, 2-arm clinical trial/Brazil	26 overweight adults, 15 ♂, 11 ♂, age 31.3 ± 8.5 y, BMI 30.4 ± 2.4 kg/m^2^	Participants were allocated to the control group (*n* = 13) or the yacon flour group (*n* = 13; consumed a breakfast drink that contained 25 g of yacon flour/6 w daily). Intestinal permeability, fecal SCFAs, oxidative stress, and inflammatory markers were evaluated in vivo.	Compared with the placebo, none of the isolated flavanol treatments significantly changed systolic or diastolic BP, plasma nitric oxide, or arterial stiffness in the yacon flour group after 2 h or 4 weeks.	Flatulence and abdominal pain (yacon flour group)
[78]	Randomized, single-blind, controlled, acute crossover/Brazil	15 volunteers, 1 ♂, 14 ♀, with fasting glycemia 87.88 ± 1.21 mg/dL, BMI 21.06 ± 0.28 kg/m^2^, body fat 23.23 ± 1.19%, age 24.87 ± 0.71 y	Participants received 350 mL of yacon shake (21 g of yacon flour with 7.4 g of FOS) or the control on 2 non-consecutive days (washout); 60 min after the test meal, subjects consumed 25 g of glucose and restricted physical activity to a minimum for the following 3 h capillary postprandial glycemia and appetitive sensation assessments.	There were no significant differences between the yacon shake group and the controls concerning glycemic control and appetitive sensations.	NR
[79]	Randomized, placebo-controlled, double-blind/Brazil	72 elderly volunteers, ? ♂, ? ♀, with fasting glycemia 87.88 ± 1.21 mg/dL, BMI 21.06 ± 0.28 kg/m^2^, body fat 23.23 ± 1.19%, age 67.11 ± 6.11 y	In total, 37 elderly people received 7.4 g of FOS daily in freeze-dried powdered yacon (FDY) with milk or juice during breakfast, and 37 older adults received a placebo.	There were no significant differences in anthropometry or intestinal transit between groups. Furthermore, there were little differences in biochemical markers for glucose and lipid metabolism in both groups.	NR

AGEs, advanced glycation ends; BMI, body mass index; BP, blood pressure; FOS, fructo-oligosaccharide; SCFA, short-chain fatty acid; sRAGE, soluble receptor for AGE; NR, not reported.

**Table 3:** Descriptive table for the biases of the selected randomized clinical trials.

Reference	Question Focus	Appropriate Randomization	Allocation Blinding	Double-Blind	Losses (<20%)	Prognostics/Demographic Characteristics	Outcomes	Intention-to-Treat Analysis	Sample Calculation	Adequate Follow-Up
[61]	Yes	Yes	Yes	Yes	No	Yes	Yes	No	No	Yes
[76]	Yes	Yes	No	No	No	Yes	Yes	Yes	No	Yes
[64]	Yes	Yes	Yes	Yes	No	Yes	Yes	Yes	No	Yes
[60]	Yes	Yes	Yes	Yes	No	Yes	Yes	No	No	Yes
[77]	Yes	Yes	Yes	Yes	No	Yes	Yes	No	No	Yes
[78]	Yes	Yes	Yes	No	Yes	Yes	Yes	Yes	No	Yes
[79]	Yes	Yes	Yes	No	Yes	Yes	Yes	Yes	No	Yes

## Data Availability

Data sharing is not applicable.
